# Factors associated with timing of umbilical cord clamping in tertiary hospital of Nepal

**DOI:** 10.1186/s13104-018-3198-8

**Published:** 2018-01-31

**Authors:** Viktoria Nelin, Ashish KC, Ola Andersson, Nisha Rana, Mats Målqvist

**Affiliations:** 1 International Maternal and Child Health, Department of Women’s and Children’s Health, Uppsala University, University Hospital, 751 85 Uppsala, Sweden; 2 United Nation’s Children’s Fund (UNICEF), Nepal Country Office, UN House, Pulchowk, Kathmandu, Nepal

**Keywords:** Clinical practice, Umbilical cord, Cord clamping, Nepal

## Abstract

**Objective:**

Delayed umbilical cord clamping (DCC) (≥ 60 s) is recognized to improve iron status and neurodevelopment compared to early umbilical cord clamping. The aim of this study is to identify current umbilical cord clamping practice and factors determining the timing of clamping in a low-resource setting where prevalence of anemia in infants is high.

**Results:**

A cross-sectional study design including 128 observations of clinical practice in a tertiary-level maternity hospital in Kathmandu, Nepal. Overall 48% of infants received DCC. The mean and median cord clamping times were 61 ± 33 and 57 (38–79) s, respectively. Univariate analysis showed that infants born during the night shift were five times more likely to receive DCC (OR 5.6, 95% CI 1.4–38.0). Additionally, infants born after an obstetric complication were 2.5 times more likely to receive DCC (OR 2.5, 95% CI 1.2–5.3), and babies requiring ventilation had a 65% lower likelihood of receiving DCC (OR 0.35, 95% CI 0.13–0.88). Despite the existence of standard protocols for cord clamping and its proven benefit, the lack of uniformity in the timing of cord clamping reveals poor translation of clinical guidelines into clinical practice.

*Clinical trial registration* ISRCTN97846009

**Electronic supplementary material:**

The online version of this article (10.1186/s13104-018-3198-8) contains supplementary material, which is available to authorized users.

## Introduction

Iron deficiency is associated with impaired neurodevelopment, which can affect an individual’s cognitive, motor, and behavioral abilities [1–3]. Globally, 43% of the children below the age of 5 are anemic and 1.5% of them are severely anemic; meaning that 273 million children were anemic in the year 2011 [4]. In Nepal, 56% of children under 5 are anemic, while 75% of children between the ages of 6–11 months are anemic [5]. Delayed umbilical cord clamping (DCC), clamping the umbilical cord ≥ 60 s after delivery, has been shown to reduce anemia in infants in this setting [6], and identified as a highly cost-effective intervention [7, 8].

Delayed cord clamping has the potential to contribute approximately 75 mg of iron, corresponding to more than an infant’s 3-month requirement [13–15]. Additionally, DCC has been shown to improve fine motor and social skill development at 4 years of age [16]. DCC also seems to be protective against motor disability in very low birth weight male infants, possibly as a result of increased blood, red cell and stem cell volumes [17].

The 2012 World Health Organization (WHO) guidelines on maternal, newborn, child, and adolescent health recommends a delay in umbilical cord clamping of 1–3 min after delivery [9]. Improving the quality of intrapartum care as well as health worker adherence to DCC protocols will be critical to reduce the burden of childhood anemia and its consequences. This is especially relevant in low-income countries like Nepal where there is an increased trend of institutional delivery; in 2014, 55% of women delivered in a health facility [10]. We therefore conducted this study to assess the health worker adherence to DCC recommendations, and to identify the factors associated with the timing of cord clamping. This study was carried out as a baseline study for further studies comparing early and late cord clamping at the same hospital [6].

## Main text

### Methods

This study was conducted at a tertiary, government-run hospital in Kathmandu, Nepal, which is a central referral hospital. In 2011, 23,155 total deliveries occurred at the hospital [11].

Deliveries occurred in three units, but observations for this study were performed at the two sites where vaginal deliveries occurred-Labor Room (LR) and the Maternal and Newborn Service Center (MNSC).

Ethical approval for this observational study was received from Nepal Health Research Council as a part of a larger study evaluating the impact of a simplified neonatal resuscitation protocol on perinatal outcomes [12]. Written consent was obtained from the hospital administrator and matron, as well as nursing in-charge in each of the delivery wards.

Observations for this cross-sectional study were completed from July 10th to September 28th, 2013 by two observers. Selection of cases for observation occurred as women were admitted to either ward for expectant vaginal delivery. Cases were chosen based on observational shifts, i.e. the presence and ability of an observer to watch each case through delivery of the infant. Observations began during the first or second stage of labor and continued until at least 1 h after delivery or until the mother was transferred from the delivery ward, whichever occurred first. To complete observations, the researchers used printed checklists to record data and a mobile phone stopwatch. The observer was present in the background and did not interfere with healthcare delivery.

A structured checklist was developed using the International Federation of Gynecology and Obstetrics (FIGO) guidelines for active management of third stage of labor (AMTSL) [13], the WHO guidelines on neonatal resuscitation [14], and other related literature [15–17] to capture the variables of interest. The checklist included 18 items relevant to the current study; see Additional file 1: Annex S1 for a list of variables and data collection methods.

No sample size calculations were completed, although the aim was to observe at least fifty cases in both wards, with the goal that after these observations, routine practices could be determined. At least 12 deliveries during each of the hospital duty shifts were observed to ensure that potential changes in staffing routines throughout the day were captured.

Data are shown as mean ± standard deviation (SD) or as median and interquartile range (IQR). Statistical analysis was done using R Commander, part of the R statistical package (Version 3.0.3/3.1.1, R Foundation for Statistical Computing, Vienna, Austria). For analysis purposes, categorical variables were created from raw or continuous variables. Variables categorized whether early cord clamping (ECC) or DCC was used (DCC was defined as ≥ 60 s); AMTSL was done or not done; the hospital shift at the time of delivery was morning (07:00–13:00), evening (13:00–19:00), or night (19:00–07:00); third stage of labor was longer than 7 min or less; maternal blood loss was more than 100 mL or not; the infant was born preterm (< 37 weeks) or not; and whether the Apgar score at 1 min was less than 7 or more. AMTSL was considered complete if an uterotonic drug was given after the infant’s delivery, controlled cord traction was completed, and uterine massage was done at least once after placental delivery. Seven minutes was chosen as a cutoff for a “longer” third stage of labor as this was the third quartile for length; likewise, 100 mL was chosen as the cutoff for “greater” maternal blood loss as this was the third quartile amount.

Subgroups were compared using independent samples t-tests or one-way analysis of variance (ANOVA) for continuous variables; normal distribution was assumed. However, a test for normal distribution was also done and a comparison of the medians using a Kruskal–Wallis test was also completed for any variable not normally distributed. Chi Square analysis was used to compare categorical data between ECC and DCC groups and/or MNSC and LR groups. Finally, univariate logistic regression was used to determine the association for receiving DCC. Differences are considered significant when p < 0.05.

### Results

**Fig. 1:**
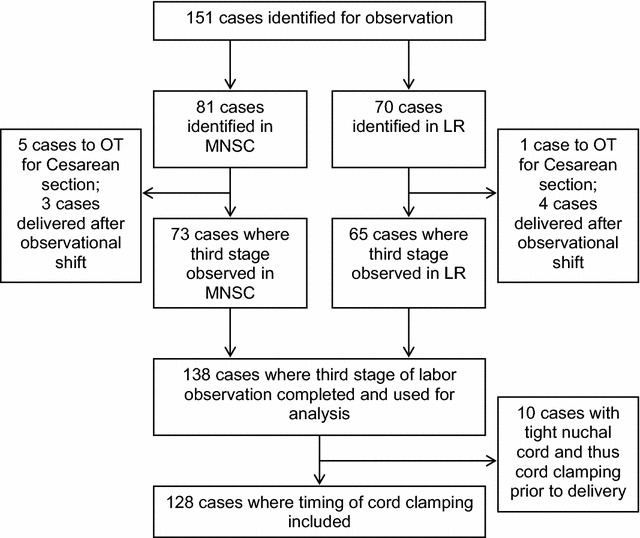
Flow chart of study participants included in this study (*MNSC* Maternal and Newborn Service Center, *LR* Labor Room, *OT* Operation Theater)

Of the 151 eligible cases, only 138 included observations of the third stage of labor, either because caesarean section was ordered or the delivery occurred after the observational shift ended. Of these 138 cases, only 128 included a measurement of timing of umbilical cord clamping, as 10 infants had tight nuchal cords (i.e. wrapped tightly around their necks), and in these cases clamping and cutting was done before the entire infant’s body was delivered (Fig. 1). Therefore, these cases were excluded from analysis. Sixty-nine cases were observed in the MNSC and 59 in the LR. All of the 128 singleton vaginal deliveries were spontaneous, with episiotomy or without. The mean gestational age at birth was 39.5 ± 1.9 weeks and twelve infants were preterm. The mean birth weight of the sample population was 2967 ± 463 grams. There were a greater number of female (77) than male (51) infants delivered (p = 0.02).

Overall, 48% of infants received DCC (≥ 60 s), 40% had cord clamping between 30–59 s, and 12% prior to 30 s. In the MNSC, the low-risk ward, 52% of infants had DCC, 41% had their cords clamped between 30–59 s, and 7% before 30 s. In the LR, the higher-risk ward, DCC was completed in 42% of cases, cord clamping occurred between 30–59 s in 39% of infants, and prior to 30 s in 19% of infants. Cord clamping was completed at 3 min or after in two cases. All infants were placed directly on the mother’s abdomen after delivery, and were therefore positioned above the perineum when clamping occurred. Cord milking was never used.

Mean and median cord clamping times among population subgroups are presented in Table 1. There was no difference in the mean cord clamping times among any of the subgroups. Timing of cord clamping was not normally distributed, so the medians of each subgroup were also compared. The median cord clamping time was significantly higher in cases where no interventions were given to the infant after delivery, as compared to those cases where infants received any intervention. In all other subgroups, median cord clamping times were similar.

**Table 1:** Median (and interquartile range, IQR) and range of cord clamping (CC) times among population subgroups

Subgroup	Median CC time in s (IQR)	p value^a^	Range of CC times in s
All deliveries	57 (38, 79)		10–189
Place of birth			
MNSC	60 (42, 74)		20–189
Labor room	53 (35, 84)	0.579	10–152
Hospital shift at delivery			
Morning (07:00–13:00)	53 (36, 84)		10–189
Evening (13:00–19:00)	55 (35, 74)		20–180
Night (19:00–07:00)	78 (64, 82)	0.056	40–145
AMTSL used			
No	61 (53, 64)		22–66
Yes	57 (37, 81)	0.969	10–189
Third stage > 7 min			
No	58 (38, 78)		10–180
Yes	54 (36, 90)	0.767	23–189
Maternal blood loss > 100 mL^b^			
No	55 (35, 80)		10–189
Yes	60 (55, 75)	0.563	25–93
Labor/delivery complications			
None	52 (38, 75)		18–180
Any	60 (35, 90)	0.378	10–189
Preterm birth^c^			
No	56 (38, 76)		10–189
Yes	68 (34, 90)	0.699	20–117
Apgar score < 7 at 1 min:			
No	60 (40, 83)		10–189
Yes	51 (35, 78)	0.104	12–152
Interventions to the infant after delivery			
None	60 (42, 82)		10–189
Any	38 (28, 60)	0.008	18–152

Labor/delivery complications included breech presentation, fetal distress, premature rupture of membranes, meconium staining, and various degrees of tears and lacerations

Interventions given to the infant included oxygen administration, penguin/bulb suction, electric suction, bag and mask ventilation, vigorous stimulation, and transfer to the Postnatal Baby Unit

*MNSC* Maternal and Newborn Service Center, *AMTSL* Uterotonic administration + CCT + Uterine massage

^a^p value determined by Kruskal–Wallis test

^b^This variable includes one missing value where maternal blood loss not recorded

^c^This variable includes two missing values where gestational age was not available

Subgroup analyses comparing the use of DCC and ECC are presented in Table 2. The use of DCC was comparable in most population subgroups. However, there were more deliveries on the night shift in the DCC group as compared to the ECC group, and infants born during the night shift were 5.6 times more likely to receive DCC than ECC (OR 5.62, 95% CI 1.38–37.96). Women with any obstetric complication during delivery had 2.5 times more likelihood of receiving DCC (> 60 s) than those who did not have any complication (OR 2.50, CI 95% 1.19–5.34). Babies requiring ventilation had a 65% lower chance of receiving DCC (OR 0.35, 95% CI 0.13–0.88) than did babies in whom ventilation was not required.

**Table 2:** Subgroup analyses for early compared to delayed cord clamping and odds ratios (OR) of delayed cord clamping (≥ 60 s) among subgroups

Subgroup	Early cord clamping % (n/N)	Delayed cord clamping % (n/N)	OR (95% CI)^a^
Labor/delivery characteristics			
Delivered in labor room (high-risk)	51 (34/67)	41 (25/61)	0.67 (0.33–1.35)
Night shift	3 (2/67)	15 (9/61)	5.62 (1.38–38.0)
AMTSL used	97 (65/67)	93 (57/61)	0.44 (0.06–2.33)
Length of third stage > 7 min	19 (13/67)	18 (11/61)	0.91 (0.37–2.23)
Maternal blood loss > 100 mL^b^	6 (4/66)	16 (10/61)	3.04 (0.95–11.6)
Obstetric complication during delivery	25 (17/67)	46 (28/61)	2.50 (1.19–5.34)
Infant characteristics			
Preterm birth^c^	7 (5/67)	12 (7/59)	1.67 (0.50–5.93)
Apgar score < 7 at 1 min	61 (41/67)	51 (31/61)	0.66 (0.32–1.32)
Any intervention to the infant	27 (18/67)	11 (7/61)	0.35 (0.13–0.88)

Labor/delivery complications included breech presentation, fetal distress, premature rupture of membranes, meconium staining, and various degrees of tears and lacerations

Interventions given to the infant included oxygen administration, penguin/bulb suction, electric suction, bag and mask ventilation, vigorous stimulation, and transfer to the Postnatal Baby Unit

Active Management of Third Stage of Labor (AMTSL) = Uterotonic administration + CCT + Uterine massage

*n* number of cases where characteristic is yes, *N* sample size

^a^Crude odds ratio from univariate logistic regression model

^b^This variable excludes one case where maternal blood loss not recorded (remaining n = 127)

^c^This variable includes two missing values where gestational age was not available

### Discussion

We found that the median time for cord clamping was 1 min and the timing of cord clamping was not associated with place of delivery, use of AMTSL, increased length of third stage of labor, increased maternal blood loss, preterm birth, or Apgar score < 7 at 1 min. DCC was more likely in cases where there was any obstetric complication and DCC was less likely in cases where the infant was given any intervention post-delivery as compared to those cases where the infants were given none.

Timing of umbilical cord clamping was not associated with the use of AMTSL thus demonstrating that DCC is not invited or enhanced by the use of AMTSL in this setting. DCC was less likely in cases where the infant received any intervention, possibly because in most cases where the infant is perceived to require assistance for stabilization the cord is clamped quickly to facilitate providing care to the infant [18]. It is interesting to emphasize that DCC was less likely in infants who received any interventions since in past randomized controlled trials examining DCC versus ECC, those infants who were asphyxiated or in need of immediate resuscitation measures were excluded due to concern for their clinical condition and health outcomes. However, the umbilical cord is not only a conduit for the transfer of blood, but also for the delivery of oxygen to the foetus/infant through the gas exchange function of the placenta.

Delayed cord clamping was used in about half of the cases at this hospital, although the wide variation in cord clamping times reveals the need for defined clinical guidelines to direct practice in this setting. Additionally, it is important to recognize potential barriers to the use of DCC and to identify factors that would help facilitate a change in clinical practice, as this is currently an evidence-based recommendation. The future studies planned at this hospital will aim to address some of the research gaps, including the use of DCC in asphyxiated infants.

## Limitations

This study was conducted at a large delivery hospital in Kathmandu in Nepal. Thus, the practices used here may differ compared to other health centers across Nepal. Due to its observational nature, there are some potential biases in this study, including intra-/inter-observer variation, as well as bias due to overt observation. This study was part of a larger one examining the implementation of the Helping Babies Breathe (HBB) protocol [19], and the observers were also involved in the HBB protocol training of some of the staff working in the delivery wards during the observational period, thus elevating the potential for desirability bias among health staff. Finally, the primary aim of this study was to describe practices, although we used logistic regression to compare groups, and our small sample size limits the statistical power of these analyses.

## Additional file

**Additional file 1.** Definitions of variables. Definitions of variables (relevant to this study) contained in the structured checklist, and their method of data collection.

## Abbreviations

AMTSL: active management of third stage of labor
DCC: delayed cord clamping
ECC: early cord clamping
FIGO: International Federation of Gynecology and Obstetrics
HBB: Helping Babies Breathe
IQR: interquartile range
LR: Labor Room
MNSC: Maternal and Newborn Service Center
OR: odds ratio
SD: standard deviation
WHO: World Health Organization
