# Supplementary material for: Factors associated with timing of umbilical cord clamping in tertiary hospital of Nepal
Source: BMC Res Notes. 2018 Jan 31;11:89. doi: 10.1186/s13104-018-3198-8 (PMC5793403; doi:10.1186/s13104-018-3198-8)
Supplement: Supplementary file 1 — Additional file 1. Definitions of variables. Definitions of variables (relevant to this study) contained in the structured checklist, and their method of data collection. [file 13104_2018_3198_MOESM1_ESM.docx]

| Outcome | Variable | Method of Data Collection |
| --- | --- | --- |
| Timing of cord clamping | - Timing of umbilical cord clamping after delivery of infant (seconds) - Positioning of the infant prior to umbilical cord clamping in relation to the perineum - Use of umbilical cord milking to increase placental transfusion | - Observation (stopwatch) - Observation checklist - Observation checklist |
| Active management of third stage of labor | - Administration of uterotonic drug (done/not done) - Timing of uterotonic administration after delivery of infant (seconds) - Type of uterotonic drug given - Route of uterotonic administration - Use of controlled cord traction (done/not done) - Use of uterine massage (done/not done at least once after delivery of placenta) - Length of third stage of labor (minutes) | - Observation checklist - Observation (stopwatch) - Observation checklist - Observation checklist - Observation checklist - Observation checklist - Observation (stopwatch) |
| Labor/delivery characteristics | - Presence of labor/delivery complications (yes/no) - Mode of delivery - Amount of maternal blood loss (milliliters) | - Observation checklist/medical records - Observation checklist/medical records - Medical records (birth provider’s reporting) |
| Infant characteristics | - Birth weight (grams) - Sex - Gestational age (weeks) - Intervention(s) given to infant after delivery (yes/no) - Apgar scores (1, 5 min) | - Medical records - Medical records - Medical records - Observation checklist/medical records - Medical records |
